# A phasing and imputation method for pedigreed populations that results in a single-stage genomic evaluation

**DOI:** 10.1186/1297-9686-44-9

**Published:** 2012-06-19

**Authors:** John M Hickey, Brian P Kinghorn, Bruce Tier, Julius HJ van der Werf, Matthew A Cleveland

**Affiliations:** 1 School of Environmental and Rural Science, University of New England, Armidale, Australia; 2 Animal Genetics and Breeding Unit, University of New England (AGBU is a joint unit of NSW DPI and the University of New England), Armidale, Australia; 3 Cooperative Research Centre for Sheep Industry Innovation, Armidale, Australia; 4 Genus plc, 100 Bluegrass Commons Blvd., Suite 2200, Hendersonville, TN, 37075, USA

## Abstract

**Background:**

Efficient, robust, and accurate genotype imputation algorithms make large-scale application of genomic selection cost effective. An algorithm that imputes alleles or allele probabilities for all animals in the pedigree and for all genotyped single nucleotide polymorphisms (SNP) provides a framework to combine all pedigree, genomic, and phenotypic information into a single-stage genomic evaluation.

**Methods:**

An algorithm was developed for imputation of genotypes in pedigreed populations that allows imputation for completely ungenotyped animals and for low-density genotyped animals, accommodates a wide variety of pedigree structures for genotyped animals, imputes unmapped SNP, and works for large datasets. The method involves simple phasing rules, long-range phasing and haplotype library imputation and segregation analysis.

**Results:**

Imputation accuracy was high and computational cost was feasible for datasets with pedigrees of up to 25 000 animals. The resulting single-stage genomic evaluation increased the accuracy of estimated genomic breeding values compared to a scenario in which phenotypes on relatives that were not genotyped were ignored.

**Conclusions:**

The developed imputation algorithm and software and the resulting single-stage genomic evaluation method provide powerful new ways to exploit imputation and to obtain more accurate genetic evaluations.

## Background

Genome-wide association studies and genetic evaluation systems using genomic information require large numbers of individuals that are both intensively phenotyped and densely genotyped for SNP across the genome to give reliable results. Dense SNP genotyping of the required number of individuals is still expensive. Therefore, research effort has focused on developing methods and strategies to impute genotypes from low-density platforms that are cheaper and easier to obtain. These imputation methods can be divided into two main categories, those that use linkage disequilibrium information (e.g. fastPHASE [1]; MaCH [2]; Beagle [3]; IMPUTE2 [4]) and those that use pedigree and linkage information e.g. [5-8]. Methods that use linkage disequilibrium information are suboptimal because they require at least some genotypes on each individual for which imputation is to be carried out. Some of the methods that use pedigree and linkage information are also less than optimal because they require all immediate ancestors to be densely genotyped. An optimal imputation strategy for practical application in breeding programs must (i) allow both ungenotyped and low-density genotyped animals to be imputed, (ii) be flexible with regard to which animals within the pedigree have high-density genotypes, (iii) use information from close and distant relatives and from close and distant SNP loci, (iv) function well in small and large datasets of moderately related individuals and (v) accurately impute genotypes for all animals in the pedigree for all SNP, including unmapped SNP.

Single-stage genomic evaluations use all available pedigree, genomic, and phenotypic information in a single analysis to estimate breeding values. This can be achieved by using a relationship matrix that combines both pedigree and genomic information (**SSMi**) Misztal et al. [9]). From an imputation perspective, SSMi has at least three potential problems. First, in its original definition, it combines two relationship matrices (pedigree and genomic) that have two different base populations definitions, although solutions for this have been proposed recently [10,11]. Second, it can be thought of as a linear imputation method e.g. [12] that ignores the particulate nature of allelic inheritance. Third, while it can give different SNP different weights in the prediction equation [13], the implementation is usually by ‘GBLUP’ - genomic best linear unbiased prediction [14], which does not use variable selection (of SNP effects), such as method BayesB [15]. An imputation method that imputes genotypes for all animals in the pedigree for all SNP provides an alternative framework for single-stage genomic evaluations that avoids these problems.

The first objective of this study was to develop a robust imputation method for pedigreed populations. The method involves simple phasing and imputation rules, long-range phasing and haplotype library imputation [16,17] as implemented in AlphaPhase1.1 [17], and segregation analysis [18]. It uses information from close and distant relatives and from close and distant SNP loci to impute genotypes for individuals for which genotype information may or may not be available, and for individuals which have close or distant relatives densely genotyped. A software package called AlphaImpute that implements the method has been made available. The second objective was to develop a single-stage genomic evaluation method (**SSAI,** standing for single-stage AlphaImpute) that is based on using the imputation method presented here to combine all pedigree, genomic, and phenotypic information and compare it to a method that ignores phenotypes for ungenotyped animals and to SSMi.

The performance of AlphaImpute in terms of both accuracy and speed was evaluated using a dataset consisting of closely related pigs from a commercial breeding line and a multiple breed dairy cattle dataset and compared to IMPUTE2 [4], a widely used software package. The performance of SSAI was evaluated within the pig dataset using the empirical accuracy of the resulting estimated breeding values. It was compared to SSMi for the complete data, and with an analysis that did not use phenotypes on ungenotyped animals.

## Methods

### Segregation analysis, long-range phasing, and haplotype library imputation

AlphaImpute combines simple phasing and imputation rules, long-range phasing, haplotype libraries, and segregation analysis to impute genotypes of all animals in a pedigree for all genotyped SNP. The program imputes alleles, when it has information to do this, and probabilities based on the algorithm of Kerr and Kinghorn [18], when the information is incomplete using single locus information. Imputed genotypes are constructed as the sum of the alleles or allele probabilities.

AlphaImpute proceeds by first separating out a set of animals that are genotyped at high-density. These animals have their SNP phased using long-range phasing and haplotype library imputation, generating haplotype libraries in the process [17]. Allele probabilities [18] are calculated for all SNP for all animals in the pedigree and alleles are imputed when these probabilities are >0.99. This can be thought of as single-locus phasing. Once the single-locus phasing and the long-range phasing are completed, the haplotypes identified in the long-range phasing are matched to alleles phased by single-locus phasing. This matching step begins with parental and other ancestral haplotypes by processing the data from the oldest to the youngest individual in the pedigree. The second part of the matching step involves processing the haplotype libraries to see if the haplotypes that an animal carries exist in the library in animals that are not identified as ancestors within the available pedigree information. The haplotype libraries are updated with any new haplotypes that are identified during this process. These steps are iterated a number of times (user defined). Finally, using the updated dataset, which now includes a large number of imputed SNP, allele probabilities are re-calculated for all SNP for all animals in the pedigree. The complete algorithm is outlined in the Appendix.

### Test datasets

The performance of AlphaImpute was tested using two real datasets, a pig and a bovine dataset that had genotype information spread sparsely across multiple generations. In these datasets, some animals had multiple generations of high-density genotyped ancestors available, while for others only some or none of the ancestors had high-density genotypes. The pig dataset (courtesy of PIC) involved a pedigree comprising 6 473 individuals, of which 3 534 were genotyped on the Illumina PorcineSNP60 BeadChip. Genotype information for chromosome 1 was used and after routine editing of the genotype data, 4 221 SNP with known genome locations remained. This population came from a single PIC breeding line and consequently the individuals were moderately to highly related to each other. The dairy cattle dataset (courtesy of LIC) involved a pedigree comprising 24 017 individuals, of which 5 057 were genotyped on the Illumina BovineSNP50 BeadChip. Genotype information for chromosome 1 was used and after routine editing of the genotype data, 2 297 SNP with known genome locations remained. This population was multiple-breed in nature, containing Holsteins, Jerseys, and crossbreds. In the pig dataset, most female and male ancestors were genotyped at high-density while in the cattle dataset only male ancestors were genotyped at high-density in most cases. In both datasets no other genotyping was used other than the low-density genotyping of the testing individuals.

The genotyped animals were divided into training and testing sets. The training set was genotyped at high-density (for all SNP), while the testing sets had different proportions of their SNP masked and then imputed. In the pig dataset, the masked proportions were 99% (**Pig99**), 95% (**Pig95**), 90% (**Pig90**), and 85% (**Pig85**). In the cattle dataset, the masked proportions were 99% (**Cat99**), 95% (**Cat95**), 90% (**Cat 90**), and 85% (**Cat85**). These densities were roughly equivalent to 500, 2 500, 5 000 and 7 500 genome-wide low-density SNP arrays. The SNP on the low-density platforms were chosen by identifying evenly spaced 5 SNP windows across the chromosome and then choosing the SNP with the highest minor allele frequency from each window. For the pig data, the testing set comprised the 509 most recently born animals that were genotyped, which reflects a scenario where candidates for selection are genotyped at low density. For the cattle data, the testing set comprised 626 randomly selected individuals across generations that had both parents known in the pedigree but not necessarily genotyped.

The accuracy of imputation of unmapped SNP was tested using the same data structures by running the program with a qualifier that does not invoke the long-range phasing and haplotype imputation steps. Therefore the only imputation carried out was based on simple single locus phasing and imputation rules and the segregation analysis procedures of Kerr and Kinghorn [18].

This study used pre-existing data sets that were recorded independently of this study, therefore no ethical approval was required.

### Imputation accuracy

The performance of AlphaImpute was compared to that of the software IMPUTE2 (Howie et al. [4]; Department of Statistics, University of Oxford, UK). IMPUTE2 was used with its default settings, and an effective population size of 1 000 was assumed. IMPUTE2 uses linkage disequilibrium information to perform imputation, requires each individual to be imputed to have some SNP genotypes and does not use pedigree information. Therefore, IMPUTE2 does not impute genotypes for ungenotyped animals in the pedigree. IMPUTE2 is a hidden-Markov model based pedigree-free imputation approach with methodological similarities to Beagle, MaCH, and fastPHASE, which have been compared to each other in several studies [4,19,20]. Generally speaking, they give similar results but Marchini and Howie [20] showed a slight advantage for IMPUTE2. Accuracy of imputation was measured as the correlation between true genotype with imputed genotype or genotype probability for reasons outlined in [21].

AlphaImpute can also impute phased alleles or provide allele probabilities for animals in the pedigree that have low-density genotypes or no genotypes. The proportion of paternal or maternal phased alleles that was called without ambiguity (i.e. alleles rather than allele probabilities were called because haplotypes could be resolved unambiguously) was also explored. For this purpose, animals in the testing set were divided into six categories depending on their pattern of relationship to their most recent densely genotyped ancestors. The categories were: both parents genotyped (**BothParents**); sire and maternal grandsire (**SireMGS**); dam and paternal grandsire (**DamPGS**); sire only (**Sire**); dam only (**Dam**); and other relatives (**Other**). The numbers of animals in each category are given in Table 1 for the pig dataset and Table 2 for the cattle dataset.

**Table 1:** Accuracy of imputation for the pig dataset

		Pig99		Pig95		Pig90		Pig85		Unmapped
Category^1^	Count^2^	AI^3^	I2^4^	AI^3^	I2^4^	AI^3^	I2^4^	AI^3^	I2^4^	AI^3^
BothParents	51	0.98	0.77	0.99	0.92	1.00	0.96	1.00	0.96	0.83
SireMGS	62	0.93	0.80	0.98	0.92	0.99	0.94	0.99	0.96	0.78
DamPGS	47	0.96	0.79	0.98	0.92	0.99	0.95	0.99	0.96	0.82
Sire	45	0.89	0.78	0.97	0.92	0.99	0.95	0.99	0.97	0.78
Dam	13	0.90	0.76	0.96	0.89	0.98	0.93	0.98	0.95	0.73
Other	291	0.86	0.79	0.94	0.91	0.97	0.95	0.97	0.96	0.76

^1^Animals in the testing set were divided into six categories depending on their pattern of relationship to their most recent densely genotyped ancestors; the categories were: both parents genotyped (BothParents); sire and maternal grandsire (SireMGS); dam and paternal grandsire (DamPGS); sire only (Sire); dam only (Dam); and other relatives (Other); ^2^Count refers to the number of individuals in each category; ^3^AI = AlphaImpute; I2^4^=IMPUTE2.

**Table 2:** Accuracy of imputation for the cattle dataset

		Cat99		Cat95		Cat90		Cat85		Unmapped
Category^1^	Count^2^	AI^3^	I2^4^	AI^3^	I2^4^	AI^3^	I2^4^	AI^3^	I2^4^	AI^3^
BothParents	28	0.97	0.64	0.99	0.92	0.99	0.94	1.00	0.95	0.85
SireMGS	224	0.87	0.60	0.97	0.91	0.98	0.95	0.99	0.96	0.82
DamPGS	7	0.92	0.63	0.97	0.87	0.98	0.91	0.98	0.95	0.84
Sire	144	0.86	0.60	0.96	0.90	0.98	0.95	0.98	0.96	0.82
Dam	4	0.95	0.63	0.98	0.90	0.99	0.97	0.99	0.95	0.85
Other	219	0.84	0.58	0.94	0.90	0.96	0.95	0.97	0.96	0.82

^1^Animals in the testing set were divided into six categories depending on their pattern of relationship to their most recent densely genotyped ancestors; the categories were: both parents genotyped (BothParents); sire and maternal grandsire (SireMGS); dam and paternal grandsire (DamPGS); sire only (Sire); dam only (Dam); and other relatives (Other); ^2^Count refers to the number of individuals in each category; _3_AI=AlphaImpute; I2^4^=IMPUTE2

^3^AlphaI = AlphaImpute.

### Single-stage genomic evaluations

Single-stage genomic evaluations use all available pedigree, genomic, and phenotypic information in a single analysis to estimate breeding values [9]. SSAI uses AlphaImpute to generate imputed or observed genotypes for all animals in a pedigree for all genotyped SNP. These genotypes can be fitted in a genomic prediction model via GBLUP [14] or a Bayesian model such as BayesB [15]. This facilitates the inclusion of the phenotypes of all animals in the pedigree in the breeding value estimation model, including those of animals that are not genotyped.

The performance of SSAI was evaluated in the pig dataset using three different growth and composition traits with heritabilities ranging from 0.38 to 0.62. This data was from the same pedigree as described previously but used SNP on all chromosomes rather than just chromosome 1. After routine editing of SNP, 52 843 SNP remained, of which 43 949 SNP were mapped to the 18 autosomal chromosomes and 8 894 SNP were unmapped. Unlike in the imputation scenarios described in the previous section, for the testing of SSAI, genotyped animals were only genotyped at high-density. The data were divided into a training and a validation set. The validation set comprised the same 509 genotyped animals from the most recent generation of the pedigree that were used for testing the imputation accuracy as described previously. These validation animals also had progeny test EBV (**ptEBV**) and accuracies from single-trait BLUP evaluations. The models for estimating ptEBV used the full PIC pedigree and all data included in a typical production run for each trait but no genomic information and also the phenotypes on the validation animals or their progeny were excluded. The mean accuracy of the ptEBV for the validation animals for each trait is given in Table 3.

**Table 3:** Performance of single-stage genomic evaluation in the pig dataset

		Correlation between ^1^gEBV and ^2^ptEBV				^3^Number of phenotypes	
^4^Trait	h^2^	^5^SC1	^6,7^SC2-SSAI	^8^SC2-SSMi	Average accuracy of ptEBV	SC1	SC2
1	0.38	0.51	0.51	0.54	0.89	2632	4650
2	0.58	0.33	0.51	0.50	0.94	2643	4784
3	0.62	0.42	0.60	0.55	0.94	2675	4840

^1^gEBV = genomic estimated breeding value; ^2^ptEBV = progeny test estimated breeding value; ^3^number of phenotypes available, as not all individuals that were genotyped were phenotyped for all traits; ^4^the traits were growth and composition traits of pigs; ^5^SC1 = only the phenotypes of the 3 025 genotyped animals that were not part of the validation population were used to train the prediction equation; ^6^SC2 = the phenotypes of the 3 025 genotyped animals that were not part of the validation population and the phenotypes of the 2 939 other animals in the pedigree that were not genotyped were used to train the prediction equation; ^7^SSAI = single stage methodology of AlphaImpute; ^8^SSMi = single stage methodology of Misztal et al. [9].

Two different training sets were used. In scenario 1 (**SC1**), only the phenotypes of the 3 025 genotyped animals that were not part of the validation population were used to train the prediction equation. In scenario 2 (**SC2**), the phenotypes of the 3 025 genotyped animals that were not part of the validation population and the phenotypes of the 2 939 other animals in the pedigree that were not genotyped were used to train the prediction equation. Not all animals in the training population had phenotypes for all traits and the numbers of phenotypes included in the analysis for SC1 and SC2 for each trait are given in Table 3.

In order to evaluate the performance of SSAI, genomic estimated breeding values (gEBV) were estimated for SC1 using the Bayesian model BayesBFast [22] implemented in AlphaBayes [23]. For SC2, gEBV were estimated using SSAI and SSMi. For SC2, SSAI was applied by first using AlphaImpute to generate imputed genotypes and gEBV were then estimated by BayesBFast. AlphaBayes was chosen because it can include real numbers in its design matrix for the SNP. This is important because, when there is insufficient information to fully impute an allele (i.e. call the allele as a 0 or a 1) in the resulting genotypes, the sum of the allele probabilities generated by AlphaImpute are real numbers between 0 and 2, rather than the more usual 0, 1, or 2 integers used for coding genotypes. An alternative Bayesian genomic selection software package that rounds probabilities to integers was also tested and did not perform as well. For SC2, SSMi was implemented as described by Aguilar et al. [24], using BLUPF90.

## Results

### Performance of AlphaImpute

#### Accuracy of imputation

Genotypes imputed with AlphaImpute had very high accuracy across all categories of animals in both datasets and accuracies were greater than with IMPUTE2 (Tables 1 and 2). The higher accuracy of AlphaImpute over IMPUTE2 increased with reducing density of the low-density panels. For both AlphaImpute and IMPUTE2, the correlation between true and imputed genotypes increased with increasing relatedness of the animal to be imputed with its most recent genotyped ancestors and as its genotype density increased. Animals that had both parents genotyped had imputation accuracies of 0.98, 0.99, 1.00, 1.00 for Pig99, Pig95, Pig90, and Pig85 for AlphaImpute, corresponding to accuracies of 0.77, 0.92, 0.96, and 0.96 for IMPUTE2. Animals that had both parents genotyped had accuracies of 0.97, 0.99, 0.99, and 1.00 respectively for Cat99, Cat95, Cat90, and Cat85 for AlphaImpute and of 0.64, 0.92, 0.94, and 0.95 for IMPUTE2.

Sires and grandsires play an important role in commercial breeding programs and it may be economically feasible to use a genotyping strategy that involves genotyping these animals at high-density and candidates for selection at low-density. For the pig population, the SireMGS category of animals had imputation accuracies of 0.93, 0.98, 0.99, and 0.99 for Pig99, Pig95, Pig90, and Pig85 for AlphaImpute and of 0.80, 0.92, 0.94, and 0.96 for IMPUTE2. For the cattle population, the SireMGS category of animals had accuracies of imputation of 0.87, 0.97, 0.98, and 0.98 for Cat99, Cat95, Cat90, and Cat85 for AlphaImpute, and 0.60, 0.91, 0.95, and 0.96 for IMPUTE2.

**Figure 1:**
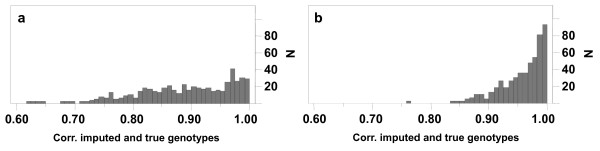
Distribution of the correlations between true and imputed genotypes for the test animals for the (a) Pig99 and (b) Pig95 scenarios.

**Figure 2:**
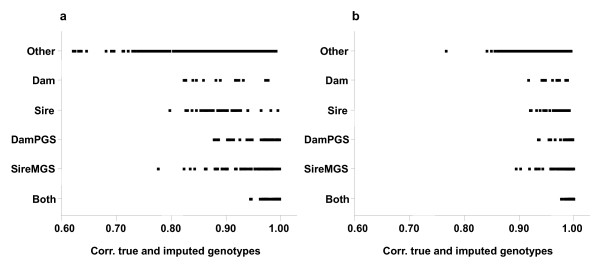
^**1**^**Correlations between true and imputed genotypes for the test animals for the (a) Pig99 and (b) Pig95 scenarios, separated by category of animals.**^1^Animals in the testing set were divided into six categories depending on their pattern of relationship to their most recent densely genotyped ancestors; the categories were: both parents genotyped (BothParents); sire and maternal grandsire (SireMGS); dam and paternal grandsire (DamPGS); sire only (Sire); dam only (Dam); and other relatives (Other).

The distribution of imputation accuracies was skewed, with most animals having very high accuracy and a small number of individuals with much lower accuracy. The distribution of imputation accuracy is shown in Figure 1 for the Pig99 and Pig95 scenarios. For Pig99, 14% of the animals had an imputation accuracy lower than 0.80 but it was never lower than 0.60. Imputation accuracy was very high for all animals that had both parents genotyped (Figure 2). Problems tended to arise for animals with no close ancestors genotyped (i.e. “Other”), especially for Pig99 (Figure 2).

**Figure 3:**
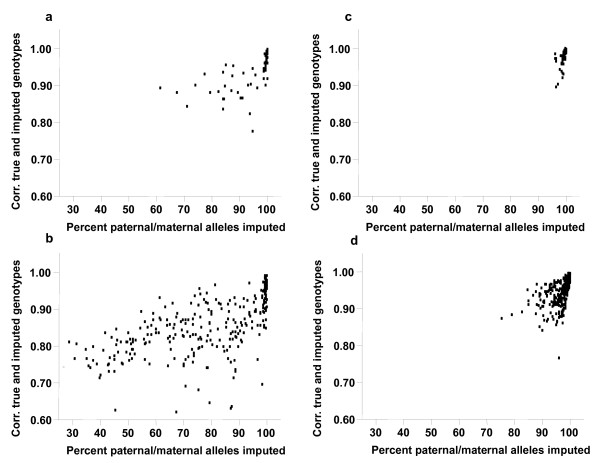
**Scatter plots of the correlation between true and imputed genotypes for the test animals and the percent of paternal or maternal alleles called without ambiguity.** (**a**) Paternal Pig99, (**b**) Maternal Pig99, (**c**) Paternal Pig95, (**d**) Maternal Pig95.

**Figure 4:**
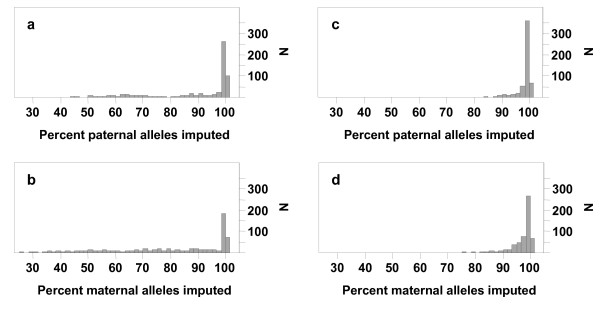
**Distribution of the percentage of paternal or maternal alleles called without ambiguity for the test animals for the Pig99 and Pig95 scenarios.** (**a**) Paternal Pig99, (**b**) Maternal Pig99, (**c**) Paternal Pig95, (**d**) Maternal Pig95.

**Figure 5:**
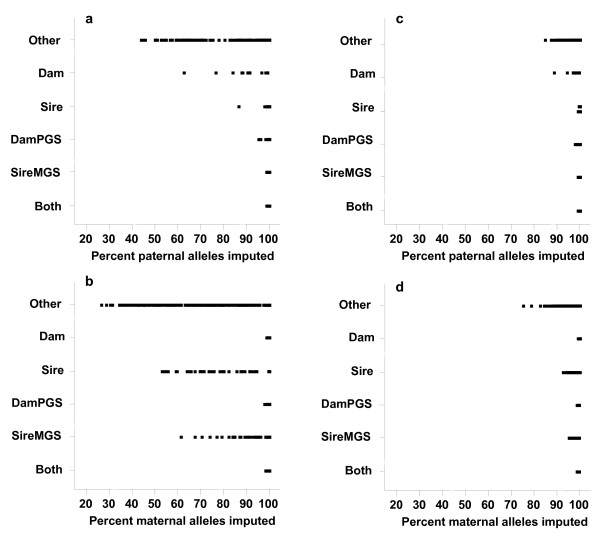
^**1**^**Percentage of imputed paternal and maternal alleles for the test animals separated by the category of the animals.** (**a**) Paternal Pig99, (**b**) Maternal Pig99, (**c**) Paternal Pig95, (**d**) Maternal Pig95. ^1^Animals in the testing set were divided into six categories depending on their pattern of relationship to their most recent densely genotyped ancestors; the categories were: both parents genotyped (BothParents); sire and maternal grandsire (SireMGS); dam and paternal grandsire (DamPGS); sire only (Sire); dam only (Dam); and other relatives (Other).

The relationship between imputation accuracy and the minimum % paternal or maternal alleles called without ambiguity is shown in Figure 3. Animals with less than 90% of their alleles called without ambiguity tended to have a lower accuracy, suggesting that a threshold could be used to give a warning about the quality of imputation for animals that are likely to have large numbers of errors or that have low relationships to animals with high-density genotypes and, hence, little information for imputation. Figures 4 and 5 show the distribution of the % alleles called without ambiguity. This % was in line with expectations given the category that an animal belonged to. For example, for individuals for which only dams were genotyped (category *Dam*), the % of maternal alleles imputed was high.

Imputation accuracy for unmapped SNP was lower than for mapped SNP, with accuracies ranging from 0.73 to 0.83 for the pig dataset (Table 1) and 0.82 to 0.85 for the dairy cattle dataset (Table 2). Within each dataset, the trend for increasing imputation accuracy with increasing levels of genotyping of immediate ancestors (i.e. parents and grandparents) that was observed for mapped SNP, was also observed for unmapped SNP. Imputation of unmapped SNP is based on single-locus phasing and imputation and segregation analysis and therefore gives an indication as to the power of this component for the purposes of imputation in comparison to the other components, which make explicit use of haplotypes.

#### Computation time

AlphaImpute has a number of options to increase its computational efficiency. When using the basic option, which performs long-range phasing and then does the imputation, it took 18 hours to carry out the imputation for the whole genome (i.e. the genotyped SNP on all 18 autosomes and the unmapped SNP) for the pig dataset on a Linux server with 16 processors and 96 GB of RAM (the RAM was not needed because memory requirements are not large). Applying the update option that uses previously phased data and therefore does not require the long-range phasing step to be re-run, decreased the computation time to six hours.

### Performance of SSAI

The use of additional phenotype information on ungenotyped relatives in the pedigree resulted in gEBV with higher accuracy than ignoring this information (Table 3). For trait 2 and trait 3, the accuracy of SC2-SSAI was 0.51 and 0.60, while for SC1 it was 0.39. For trait 1, there was no difference in the accuracy of gEBV. However, the accuracy was already high (0.51) for this trait. There was little overall difference in the accuracy of gEBV estimated from SC2-SSAI and SC2-SSMi. The computational time required for SSAI was greater than for SSMi because SSMi did not require AlphaImpute to be run.

## Discussion

AlphaImpute is a flexible tool that imputes genotypes and alleles accurately and quickly for datasets with large pedigrees and large numbers of genotyped markers. When pedigree information is available, it is more robust to data structure in terms of density of the low-density genotyping platform and relationships among animals than the widely used software IMPUTE2. The performance of IMPUTE2 dramatically dropped off as the complexity of imputation increased (i.e. lower relationships to high density animals, lower density genotyping). In this study, imputation by AlphaImpute was very accurate for two datasets that had very different features. The pig dataset comprised a group of highly related animals from a single line, whereas the cattle dataset was multiple-breed in nature but with groups of highly related animals within each breed. The pig dataset had many more females than males genotyped, while in the cattle dataset, most of the genotyped animals were male. In the pig dataset, the animals to be imputed came from the most recent generation but in the cattle dataset they were selected at random from across the pedigree. AlphaImpute has also been used in several commercial datasets in multiple species (data not shown). Almost without exception it gives results that are at least as good as the results presented in this study. The computational time required for some of these other datasets illustrates the computational cost for a wide variety of scenarios. Imputing a whole genome in a poultry pedigree of 18 000 birds took similar amounts of time (Andreas Kranis, personal communication) to that required for the pig dataset in this study (circa 18 hours for the full algorithm). Imputing using the full algorithm on a small dataset of 1500 animals took two hours, while the update option reduced this to 15 minutes. Imputing one chromosome for a pedigree of 300 000 animals was not feasible (Jarmilla Johnston, personal communication) and may require parallelizing the imputation steps. The numbers of animals, the depth of the pedigree, and the relationships between genotyped animals affect computation time.

Most imputation methods include two steps, a phasing step that involves resolving the haplotypes of high-density genotyped animals, and an imputation step that involves identifying which combination of these haplotypes match the low-density genotyped animals or ungenotyped animals that have allele probabilities. The phasing step is the most difficult component. AlphaImpute uses the long-range phasing and haplotype library imputation capacity of AlphaPhase1.1 [17] to phase genotypes. This approach allows great flexibility in the pedigree structure among high-density genotyped animals. Multiple generations of high-density genotyped animals within constrained pedigree structures, such as having both parents being genotyped and large half sib family groups e.g. [6], are not needed. The high-density genotyped animals can be scattered across a pedigree, or all be from the same generation or have no recent pedigree links. Unmapped SNP are imputed using the algorithm of Kerr and Kinghorn [18] on a single-locus basis without using haplotype information.

To tackle the problem of imputation, AlphaImpute combines long-range phasing, haplotype library imputation, simple phasing and imputation rules, and segregation analysis. It accesses and updates all this information by iterating across each gamete by accessing haplotypes of varying length and varying starting and ending positions, and passes down the pedigree, and through the haplotype libraries, eliminating ancestral or other haplotypes to accumulate evidence about the alleles that an individual carries at each SNP. It iterates this whole process a number of times before finally using segregation analysis [18] to re-calculate probabilities for alleles that are still missing. In a previous attempt at imputation [25,26], all genotype probabilities (i.e. without a truncation at >0.99) were used to give a score for the match of each pair of haplotypes to the single-locus phasing information. This approach was impaired by the large number of haplotype pairs available and the resulting very high selection intensity for choosing the best-fitting haplotype pair with many haplotype combinations that have similar scores by random chance. Hence this approach was not used here.

AlphaImpute provides phased alleles or allele probabilities for all genotyped SNP for all animals in the pedigree and resolves parent of origin of all alleles along each chromosome. Therefore, it can be used as an accurate haplotyping program when pedigree information is available. This allows interesting applications, such as modeling identity by descent probabilities for estimation of gEBV and QTL detection, or models where parent of origin is relevant, such as models with imprinting or crossbreeding effects [27].

### Strategy for routine use of AlphaImpute

AlphaImpute was designed for routine use in commercial breeding programs that carry out imputation as often as weekly. In these circumstances, the data flow may be such that large numbers of new low-density genotyped animals are added weekly while new high-density genotyped animals may only be added every few weeks or months. The program has an update option for such a situation. This update option involves only phasing the high-density animals whenever significant numbers of new high-density genotyped animals are added. This dramatically reduces the computation time for the weekly imputation runs. Further options to increase flexibility of use include an ability to read in phased alleles from other haplotyping software (e.g. half-sib family haplotyping software [28]) under the condition that the parental origin of each phased allele is known, and to select a subset of the high-density genotyped individuals to have their haplotypes resolved by long-range phasing and haplotype library imputation. For example, this subset could be the key sires in a pedigree or it could be a subset of animals that could not have their phase resolved by half-sib family haplotype phasing.

### SSAI and increasing the accuracy of genomic selection

Single-stage genomic evaluations [9] are appealing because they use all available pedigree, genomic, and phenotypic data, and may avoid bias that could be introduced by two-stage genomic selection strategies [29]. Both SSAI and SSMi increased the accuracy of the gEBV compared to the base scenario SC1 but SSAI did not outperform SSMi. SSAI uses a single base for all animals in the pedigree, which is determined by the allele frequencies in the data. In contrast, with SSMi the base is not necessarily the same for genotyped and pedigreed animals (although alternatives have been proposed [10,11] based on ideas of Powell et al. [30]). For the data analyzed here, the differences between these base populations may not have been large enough to result in dramatic differences in performance. The other difference between the methods was that SSMi used GBLUP, assuming equal variance at all SNP, whereas SSAI used a Bayesian method (BayesB) that allows for unequal variances for each SNP. BayesB has shown little benefit over GBLUP models if the traits are more polygenic in nature e.g. [31]. Finally, with millions of genotyped animals, SSMi may become computationally infeasible because it requires full inversion of two very dense matrices for the genotyped animals. SSAI does not require explicit inversion of any matrices and use of parallelized computation may make it a feasible method for datasets with millions of genotyped or imputed animals. Other solutions for single-stage methodology avoid the need for inversion are under development e.g. [Legarra A, Ducrocq V: Computational strategies for national integration of phenotypic, genomic and pedigree data in a single-step BLUP, submitted].

One weakness of SSAI may be that the genotypes imputed will not be very different from two times the allele frequency (i.e. no information for imputation) for animals that are very distantly related to animals that are genotyped, or when only a small proportion of the animals in a dataset have genotype information. Given the prevalence of genotyping candidates for selection in breeding programs incorporating genomics, these distantly related and ungenotyped animals would contribute little information to the estimated breeding value of candidates for selection. However, if their phenotypes are useful to increase contemporary group sizes or provide contrasts with which breeding values can be estimated, an optimal solution may be to use a joint SSAI/SSMi approach. The SSMi approach automatically accounts for the uncertainty in imputation, which increases with decreasing relationship between genotyped individuals and individuals that are to be imputed [32,33]. Thus, there is a trade off between the more accurate imputation with SSAI and the more appropriate way of accounting for imputation error in SSMi.

### Availability

Both AlphaImpute and AlphaBayes were written in Fortran 95 and are available for research purposes from http://sites.google.com/site/hickeyjohn.

## Conclusion

A method and software package for phasing and imputation was developed for pedigreed populations and resulted in much better accuracies of imputed genotypes than IMPUTE2. The proposed method is fast, accurate, is robust to the pedigree structure of the genotyped animals, and can scale to large datasets. The data produced by the algorithm can be used directly in a single-stage genomic evaluation that combines all pedigree, genotypes and phenotypes in a single analysis.

## **Competing interests**

The authors declare that they have no competing interests.

## **Authors’ contributions**

JMH conceived the original ideas, designed the algorithm, and wrote the first version of the program under the supervision of BPK, BT, and JHJW. MAC contributed to the development of the ideas, algorithm, and program. JMH and MAC performed the analysis. JMH wrote the first draft of the paper. All authors read and approved the final manuscript.

## Appendix A - Description of the AlphaImpute algorithm

There are three primary steps in AlphaImpute, (1) segregation analysis to calculate allele probabilities for each locus of each animal in the pedigree, (2) long-range phasing and haplotype library imputation to phase all high-density genotyped individuals and create a haplotype library for each genomic region for the dataset, and, (3) impute missing alleles by matching the allelic probabilities to haplotypes in the haplotype library.

This algorithm is designed to work for biallelic SNP. SNP genotypes are coded as 0, 1, 2, or 3, where 0 is a homozygote, 1 heterozygote, 2 the alternative homozygote, and 3 is a missing genotype. SNP alleles are coded as 0 or 1.

**Step 1.** Using the algorithm of Kerr and Kinghorn (1996), calculate allele probabilities for each locus of each individual in the pedigree, using all pedigree and genotype information (both high and low density).

**Step 2.** Using the LRPHLI algorithm of Hickey et al. (2011) as implemented in AlphaPhase1.1, phase the individuals genotyped at high density a number of times and place the identified haplotypes in a library. LRPHLI divides chromosomes into cores of specified length (e.g. 100 SNP). By running LRPHLI a number of times, overlaps between cores are created and each locus is phased as part of different cores. This facilitates the identification of phasing error.

**Step 3.** Impute missing alleles by matching alleles imputed at **Step 1** to haplotypes phased at **Step 2**. This involves several sub-steps. These can be divided into major and minor sub-steps. Each sub-step is sequentially passed through; after each major sub-step, each minor sub-step is sequentially passed through. The description of **Step 3** will begin with a description of the minor sub-steps, followed by a description of the major sub-steps.

**Minor sub-step 1.***Parent homozygous fill in.* Fill in the allele of an offspring of a parent that has both its alleles filled in and has a resulting genotype that is homozygous.

**Minor sub-step 2.***Phase complement.* If the genotype at a locus for an individual is known and one of its alleles has been determined, then impute the missing allele as the complement of the genotype and the known phased allele.

**Minor sub-step 3.***Impute parents from progeny complement.* If one of the parental alleles is known and the other missing, then fill in the missing allele in the parent if at least one of its offspring is known to carry an allele that does not match the known allele in the parent. (e.g. if a sire has a 0 as one of its alleles and the other allele is missing but one of its offspring carries a 1 in the gamete received from the sire, then we can determine that the sire’s missing allele is 1).

**Minor sub-step 4.***Make genotype.* Any individual that has a missing genotype but has both alleles known, has its genotype filled in as the sum of the two alleles.

**Major sub-step 1.***Convert allele probabilities to phase.* Alleles with probabilities greater than 0.99 of being 0 or 1 are imputed.

**Major sub-step 2.***Fill in base animals.* If a base animal has high-density genotype information, it is filled in by arbitrarily assigning one of its haplotypes for one of its cores as coming from its paternal gamete and the other haplotype as coming from its maternal gamete. Haplotypes at other cores are appended to the central haplotypes where overlapping information can be used to determine which haplotype at an adjacent partially overlapping core matches the arbitrarily labeled paternal (maternal) haplotype.

**Major sub-step 3.***Candidate haplotype library imputation of alleles.* For each core of each round of the LRPHLI algorithm, all haplotypes that have been found and stored in the haplotype library are initially considered to be candidates for the true haplotype that an individual carries on its gametes. Within the core, all alleles that are known are compared to corresponding alleles in each of the haplotypes in the library. Haplotypes that have a number of disagreements greater than a small error threshold have their candidacy rejected. At the end of this loop, the surviving candidate haplotypes are checked for locations that have unanimous agreement about a particular allele. For alleles with complete agreement, a count of the suggested allele is incremented. Alleles are imputed if, at the end of passing across each core and each round of the LRPHLI algorithm, the count of whether the alleles are 0 or 1 is above a threshold in one direction and below a threshold in the other. This helps to prevent the use of phasing errors that originate from LRPHLI.

**Major sub-step 4.***Imputation based on parental phase.* This is similar to **Major sub-step 3,** with the exception that the process is restricted to individuals that have parents with high-density genotype information and the candidate haplotypes for each individual’s gametes are restricted to the two haplotypes that have been identified for each of its parents by the LRPHLI algorithm. Errors in phasing are accounted for in the same way as in **Major sub-step 3**.

**Major sub-step 5.***Individual phase imputation.* This is similar to **Major sub-step 3,** with the exception that the process is restricted to individuals that have high-density genotype information and the candidate haplotypes are restricted to the two haplotypes that have been identified for the individual by the LRPHLI algorithm. Effectively, it determines the parental origin of each of these haplotypes. Errors in phasing are accounted for in the same way as in **Major sub-step 3**.

**Major sub-step 6.***Internal candidate haplotype library imputation of alleles.* This step is similar to **Major sub-step 3,** with the exception that haplotype libraries are internally built using the information that has been previously imputed. Several different core lengths are used to define the length of the haplotypes and to ensure that errors can be accounted for in the same way as in **Major sub-step 3**.

**Major sub-step 7.***Internal imputation based on parental phase.* This step is similar to **Major sub-set 4,** with the exception that it is attempted for all animals in the pedigree on the basis that their parents may now have imputed high-density information. In the same way as **Major sub-step 6**, several core lengths are used to define the length of the haplotypes and to ensure that errors can be accounted for in the same way as in **Major sub-step 3**.

**Major sub-step 8.***Imputation based on identifying where recombination occurs during inheritance from parent to offspring.* Each gamete of an individual is examined from the beginning to the end and from the end to the beginning of the chromosome. In each direction, at loci where both the individual and its parent are heterozygous and have phase information resolved, this information is used to determine which of the parental gametes the individual received. Loci for which this cannot be determined but which are between two loci that (a) can be determined and (b) come from the same parental gamete, are assumed to come from this gamete (i.e. no double recombination event in between). Alleles are imputed in the individual when analysis in both directions of the chromosome has identified the same inherited gamete and when the parent is phased for this locus in the suggested gamete, subject to the restrictions that the number of recombination events for the individuals is less than a threshold and that the region in which two recombination events occurred exceeds a threshold length. **Major sub-step 8** is iterated a number of times with increasingly relaxed restrictions. After each iteration, the minor sub-steps are also carried out.

**Major sub-step 9.***Recalculate genotype probabilities*. Using the imputed genotype information, the allele probabilities and genotype probabilities are recalculated as in **Step 1**. Alleles that are still missing are imputed as the recalculated allele probability. Missing genotypes are imputed from the allelic probabilities when both alleles are still missing, in advance of the recalculation of allele probabilities, or from the imputed allele and the allele probability when only one allele was not imputed.

### Other miscellaneous steps

Steps are also included to divide the data into the high and low density sets of animals, to edit the SNP data, check for Mendelian inconsistencies, and identify base parents.
